# Analysis of the brain mural cell transcriptome

**DOI:** 10.1038/srep35108

**Published:** 2016-10-11

**Authors:** Liqun He, Michael Vanlandewijck, Elisabeth Raschperger, Maarja Andaloussi Mäe, Bongnam Jung, Thibaud Lebouvier, Koji Ando, Jennifer Hofmann, Annika Keller, Christer Betsholtz

**Affiliations:** 1 Department of Immunology, Genetics and Pathology, Rudbeck Laboratory, Uppsala University, Uppsala, Sweden; 2 Integrated Cardio Metabolic Centre (ICMC), Karolinska Institute, Novum, SE-141 57 Huddinge, Stockholm, Sweden; 3 Division of Neurosurgery, Zürich University Hospital, Zürich University, Zürich, Switzerland

## Abstract

Pericytes, the mural cells of blood microvessels, regulate microvascular development and function and have been implicated in many brain diseases. However, due to a paucity of defining markers, pericyte identification and functional characterization remain ambiguous and data interpretation problematic. In mice carrying two transgenic reporters, *Pdgfrb-eGFP* and *NG2-DsRed,* we found that double-positive cells were vascular mural cells, while the single reporters marked additional, but non-overlapping, neuroglial cells. Double-positive cells were isolated by fluorescence-activated cell sorting (FACS) and analyzed by RNA sequencing. To reveal defining patterns of mural cell transcripts, we compared the RNA sequencing data with data from four previously published studies. The meta-analysis provided a conservative catalogue of 260 brain mural cell-enriched gene transcripts. We validated pericyte-specific expression of two novel markers, vitronectin (*Vtn*) and interferon-induced transmembrane protein 1 (*Ifitm1*), using fluorescent *in situ* hybridization and immunohistochemistry. We further analyzed signaling pathways and interaction networks of the pericyte-enriched genes *in silico*. This work provides novel insight into the molecular composition of brain mural cells. The reported gene catalogue facilitates identification of brain pericytes by providing numerous new candidate marker genes and is a rich source for new hypotheses for future studies of brain mural cell physiology and pathophysiology.

All vertebrate blood vessels contain two principal cell types, endothelial cells (EC) and mural cells. The mural cells of larger blood vessels, vascular smooth muscle cells (VSMC), are contractile and regulate vascular tone and blood flow. Less is known about the microvascular mural cells, a.k.a. pericytes (PC)^1^,^2^,^3^, but their importance in vascular development, maintenance and pathology, in the central nervous system (CNS) in particular, is increasingly discussed. Mouse studies demonstrate that PC are necessary for vascular morphogenesis and EC quiescence in the brain and retina^4^,^5^,^6^,^7^,^8^ and for the formation of an intact blood-brain barrier (BBB)^9^,^10^. In addition, PC are implicated in regulation of cerebral blood flow^11^,^12^, although the identity of the contributing mural cell remains a topic of debate^13^. PC also regulate the localization of certain proteins to astrocyte end feet^9^. However, the PC-derived signals involved in these processes are unknown. PC have also been implicated in the pathogenesis of several human brain diseases^14^. For example, PC death or damage has been observed in diabetic retinopathy^15^,^16^, brain ischemia and stroke^11^, traumatic brain injury^17^,^18^,^19^,^20^, septic encephalopathy^21^,^22^, and in genetic brain diseases, including primary familial brain calcification (PFBC)^23^,^24^, early-adult onset dementia^25^ and cerebral proliferative glomeruloid vasculopathy^26^. Reduced or altered PC have been reported in Alzheimer’s disease (AD)^27^,^28^,^29^,^30^,^31^,^32^, mild dementia^33^ and amyotrophic lateral sclerosis (ALS)^34^,^35^. The PC phenotype may be secondary in many of these conditions. An active involvement in disease pathogenesis has been suggested in mice expressing the AD-risk allele *APOE4*, where disruption of BBB integrity depends on MMP9 expression by PC^36^. Direct involvement of PC in PFBC pathogenesis is also plausible, since disease-causing mutations have been reported in the platelet-derived growth factor B (PDGFB) and PDGF receptor-beta (PDGFRB) genes^23^,^37^, which have known roles in PC biology^38^.

The notions that PC are abundant in the CNS and play important roles in cerebrovascular biology have stimulated efforts to molecularly characterize these cells using different profiling methods. However, PC are difficult to separate from EC, and therefore, mostly indirect methods have been used to deduce the PC transcriptome^9^,^39^,^40^. Some information is also available through brain single cell-sequencing projects^41^. We reasoned that by comparing published data with direct profiling of isolated brain mural cells, we would derive significantly deeper and more authentic information on the brain mural cell transcriptome. To this end, we generated a double transgenic mouse by crossing mice expressing enhanced green fluorescent protein (eGFP) under control of the *Pdgfrb* promoter (Gensat.org) with mice expressing DsRed under control of the *NG2* promoter^42^. The double reporter mice (*Pdgfrb-eGFP/NG2-DsRed* mice) expressed both reporters only in mural cells in the brain, whereas the single reporters were also expressed in other neuroglial cells in non-overlapping patterns. This allowed us to isolate brain mural cells by FACS and analyze the cells with high throughput next-generation RNA sequencing (RNA-seq).

We combined the results from the four published studies^9^,^39^,^40^,^41^ with our newly produced RNA-seq data into a meta-analysis with the aim of constructing a catalogue of brain mural cell-*enriched* genes. The resulting gene catalogue provides direct insights into the unique molecular composition, signaling pathways and protein interaction networks within vascular mural cells of the brain.

## Results

### Specific labeling of mural cells in *Pdgfrb-eGFP/NG2-DsRed* mice

**Figure 1:**
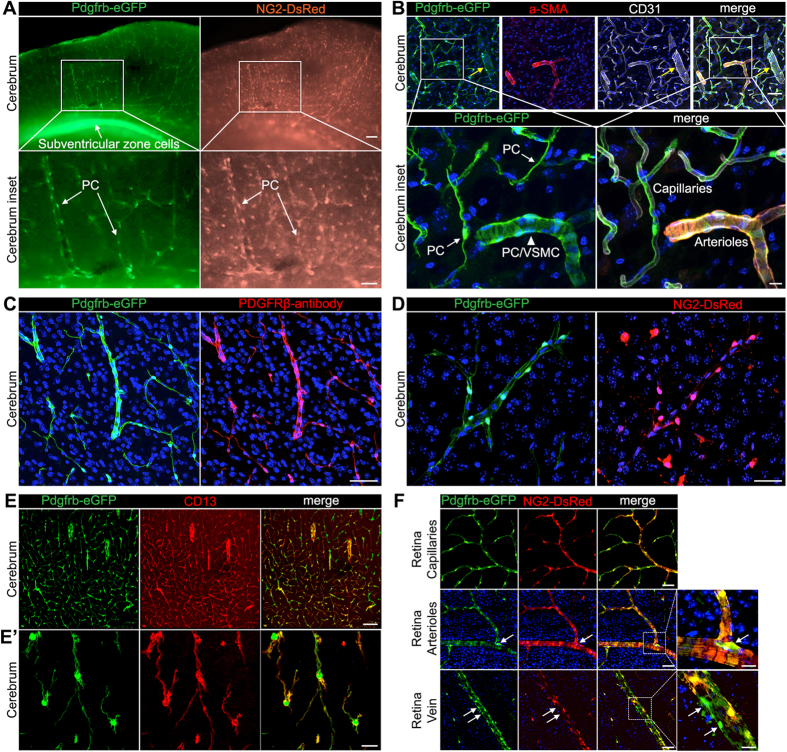
Specific labeling of central nervous mural cells in *Pdgfrb-eGFP/NG2-DsRed* mice. (**A**) Expression pattern of *Pdgfrb-eGFP* and *NG2-DsRed* in cerebrum of *Pdgfrb-eGFP/NG2-DsRed* mice. In the lower panels, cells lining capillaries and with concurrent expression of both signals are pericytes (PC). The scale bars in the top and lower panels are 100 μm and 50 μm, respectively. (**B**) *Pdgfrb-eGFP* positive cells line blood vessels visualized by CD31 staining (in white) in the cerebrum of *Pdgfrb-eGFP* mice. Some of eGFP expressing cells express also alpha-smooth muscle actin (α-SMA, in red). The white arrows and arrowhead in insert point to the PC in the capillaries and PC/VSMC in the arteriole, respectively. Yellow arrows point to the vein covered by eGFP positive cells. The scale bars in top and lower panels are 50 μm and 10 μm, respectively. (**C**) *Pdgfrb-eGFP* positive cells in the cerebral cortex of *Pdgfrb-eGFP* mouse are expressing PDGFRβ (in red). The scale bar is 50 μm. (**D**) *Pdgfrb-eGFP* and *NG2-DsRed* expression in cerebral capillaries of *Pdgfrb-eGFP/NG2-DsRed* mice. The *Pdgfrb-eGFP*-positive cells surrounding capillaries are also positive for NG2-DsRed. The scale bar is 30 μm. (**E**) *Pdgfrb-eGFP* expressing cells are positive for PC marker CD13 (in red). E’ shows a higher magnification image. The scale bars in E and E’ are 100 μm and 10 μm, respectively. (**F**) Expression pattern of *Pdgfrb-eGFP* and *NG2-DsRed* in the retina of *Pdgfrb-eGFP/NG2-DsRed* mice. White arrows indicate *Pdgfrb-eGFP*-positive cells with weak or undetectable *NG2-DsRed* expression. The scale bars in left panels and the right enlarged panels are 50 μm and 10 μm, respectively.

We crossbred *NG2-DsRed* mice^42^ with *Pdgfrb-eGFP* mice (Gensat.org. Line name: Tg(Pdgfrb-eGFP)JN169Gsat/Mmucd) with the assumption that simultaneous expression of the two markers in the same cells would allow specific identification and enrichment of PC. *Pdgfrb* and NG2 are two commonly used markers for mural cells, neither of which are completely PC-specific. Their non-mural cell expression in the brain appeared non-overlapping, however, (Fig. 1A,D) with abundant *Pdgfrb-eGFP* expression noticeable in the subventricular zone and *NG2-DsRed* expression scattered throughout the brain in cells that likely correspond to oligodendrocyte progenitors (OPC)^43^. Cells with concurrent expression of *Pdgfrb-eGFP* and *NG2-DsRed* were invariably associated with the blood vessel endothelium, and had the expected location, density, distribution and morphology of vascular mural cells (Fig. 1A). To characterize these cells in more detail, we immuno-stained brain sections from *Pdgfrb-eGFP* mice using antibodies against alpha-smooth muscle actin (α-SMA) or CD31 (PECAM1) (Fig. 1B). This revealed that *Pdgfrb-eGFP*-positive cells occupied all types of blood vessels, i.e. arteries, veins and capillaries, as defined by their morphology and diameter^2^. In arteries, *Pdgfrb-eGFP*-positive cells co-labeled with α-SMA and displayed the typical morphology of VSMC, suggesting that they represent arterial and arteriolar VSMC (Fig. 1B, inserts). *Pdgfrb-eGFP*-positive cells lining smaller veins and capillaries expressed very low, or undetectable, levels of α-SMA, as revealed by antibody staining, and displayed the characteristic morphology of venous VSMC and PC, respectively (Fig. 1B). In separate stainings, we confirmed that the *Pdgfrb-eGFP*-positive cells were labeled with antibodies against PDGFRβ (Fig. 1C) and CD13 (Fig. 1E), supporting their mural cell identity, and that the *Pdgfrb-eGFP*-positive cells surrounding capillaries were also positive for *NG2-DsRed* (Fig. 1D). Finally, we explored the overlap between *Pdgfrb-eGFP* and *NG2-DsRed* expression in different types of vessels in different parts of the CNS, including the retina (Fig. 1F and data not shown). Whereas we found a complete overlap between *Pdgfrb-eGFP* and *NG2-DsRed* expression in capillary PC, and little if any variation in marker expression between individual PC, the expression in arteriolar and venular mural cells was more heterogeneous. In particular, we noticed that some strongly *Pdgfrb-eGFP*-positive cells in arteriolar and venular walls had weak or undetectable *NG2-DsRed* expression (Fig. 1F, white arrows). The location of these cells did not display any particular bias, and we therefore do not know if this heterogeneity reflects the existence of different subtypes of mural cells, or stochastic dynamic fluctuations of the marker expression. However, we conclude, based on extensive immunohistological characterization, that double *Pdgfrb-eGFP/NG2-DsRed* positive cells comprise both capillary PC and arteriolar and venular mural cells and likely do not represent other cell types in the brain.

### RNA-seq of brain mural cells and microvascular fragments

**Figure 2:**
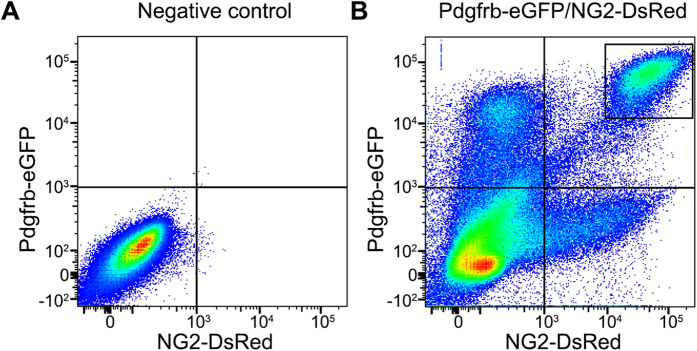
FACS of mural cells from brains of *Pdgfrb-eGFP/NG2-DsRed* mice. Dot blots showing the intensity of eGFP and DsRed channel of analyzed cells from (**A**) a negative control (C57BL6) and (**B**) *Pdgfrb-eGFP/NG2-DsRed* mice. The sorted, double-positive cell population is indicated by the rectangle in the upper right quadrant.

We sorted double positive cells from the brain of *Pdgfrb-eGFP/NG2-DsRed* transgenic mice using FACS (Fig. 2). RNA was extracted and sequenced by next-generation sequencing. We also determined the transcriptome of brain microvascular fragments (containing both endothelial cells and mural cells) isolated by mechanical tissue disintegration, collagenase digestion and immune-panning using anti-CD31 antibodies coupled to magnetic beads^9^. The RNA-seq data was aligned to the mouse reference genome and sequence counts were summarized. The results showed that mRNA transcripts representing 856 different genes were enriched more than two-fold in FACS isolated *Pdgfrb-eGFP/NG2-DsRed* double positive cells compared with whole microvascular fragments (False Discovery Rate < 0.05) (Supplemental Table S1).

We examined the expression levels and fold enrichment of a set of known PC markers (Table 1) in *Pdgfrb-eGFP/NG2-DsRed* double positive cells compared with microvascular fragments. Based on the FPKM values (Fragments Per Kilobase Of Exon Per Million Fragments Mapped), these markers ranged in their expression from high (*Rgs5* FPKM >1000), to medium/low abundance (*Anpep, Des, Dlk1* FPKM < 100 (Table 1). When comparing the fold enrichment for the known PC markers between sorted cells and vascular fragments, we found that the average enrichment was approximately 9-fold (Table 1), suggesting that approximately one out of nine cells in the microvascular fragments represent PC or other mural cells.

### Identification of brain mural cell-enriched gene transcripts

We compared the RNA-seq results with the results from four previously published studies in which mural cell transcriptome data has been reported (Table 2). These studies include two microarray analyses of microvascular fragments isolated from PC-deficient mouse mutants, which identified 453 and 135 significantly down-regulated gene transcripts, respectively, in the PC-deficient states^9^,^39^. The down-regulated transcripts would be expected to include PC-enriched transcripts but also EC transcripts positively regulated by PC. A third study also used an indirect approach, in which EC isolated from Tie2-GFP transgenic mice were compared before and after panning using anti-PDGFRβ antibodies to remove contaminating PC^40^. This procedure revealed 175 genes as putatively brain PC-enriched and 1631 genes as brain EC-enriched. The fourth study, a single cell RNA-seq of brain tissue^41^, revealed 155 genes to be predominantly enriched in brain vascular mural cells. A comparison between the five studies (the present and previous four) is provided in Table 2. Mentioned gene lists are listed in Supplementary Table S1.

Since PC and EC are embedded within a common microvascular basement membrane (BM), these cells are difficult to separate and isolate without any reciprocal contamination. In order to subtract contaminating EC-specific transcripts from the 5 mural cell datasets (Table 2), we made use of the brain EC-enriched gene list (1631 genes) reported by Daneman *et al*.^40^. Using this approach, the degree of contamination of EC-enriched transcripts in the 5 datasets ranged from 6% to 27% (Table 2).

### Integration of five different datasets to reveal a brain mural cell transcriptome

**Figure 3:**
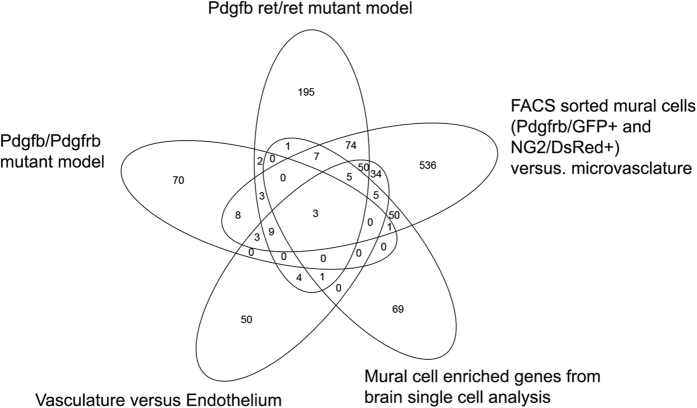
Venn diagram showing the overlap of identified mural cell genes between different datasets. The studies where these datasets have been reported are listed in Table 2.

A comparison between the results obtained from the five different approaches to identify brain mural cell-enriched transcripts is illustrated in a Venn diagram (Fig. 3). A surprisingly limited overlap was noticed, and, moreover, each approach identified a substantial number of mural cell-enriched genes not supported by the other datasets. Only three transcripts (myosin light polypeptide 9 regulatory (*Myl9*), myosin light polypeptide kinase (*Mylk*), solute carrier family 19 member 1 (*Slc19a1*) were commonly identified by the five studies. Of these three genes, *Mylk* and *Myl9* are involved in the signaling pathways regulating VSMC contraction, and they are usually co-regulated in tissue transcript profiling experiments, as exemplified in cancer^44^. Brain single cell RNA sequencing showed that *Mylk* and *Myl9* are both abundantly expressed in VSMC^41^. *Slc19a1* is a folate transporter, and while it is supposed to be broadly expressed (with high levels in e.g. liver and lung)^45^, its role in brain mural cells has not been explored previously to our knowledge. Interestingly, the common identification of *Myl9, Mylk* and *Slc19a1* in the five studies could not be explained by high mRNA abundance, which ranged between FPKM≈600 (*Myl9*) to FPKM≈100 (*Slc19a1*).

In total, 1180 different genes were identified as enriched in mural cells by at least one study. For our further analyses, we focused on genes that were identified by at least two studies, hence those that could be regarded as cross-validated and, therefore, relatively reliable. By this criterion, 260 genes were selected to be included into a brain mural cell-enriched gene catalogue (Supplementary Table S2).

These 260 genes include all known PC markers, including *Pdgfrb, Cspg4, Abcc9, Kcnj8, Rgs5, Notch3, Zic1, Dlk1, Cd248, Des, Acta2 and Anpep*. We therefore conclude that this catalogue is valuable resource for the further identification of mural cell markers, and genes that are functionally important for the biology of brain vascular mural cells, including PC.

In order to visualize the results from different studies and their comparisons, we created a website in which the brain mural cell transcriptome can be explored (http://betsholtzlab.org/brainMural/genes.html).

### Novel brain mural cell markers verified by *in situ* hybridization

**Figure 4:**
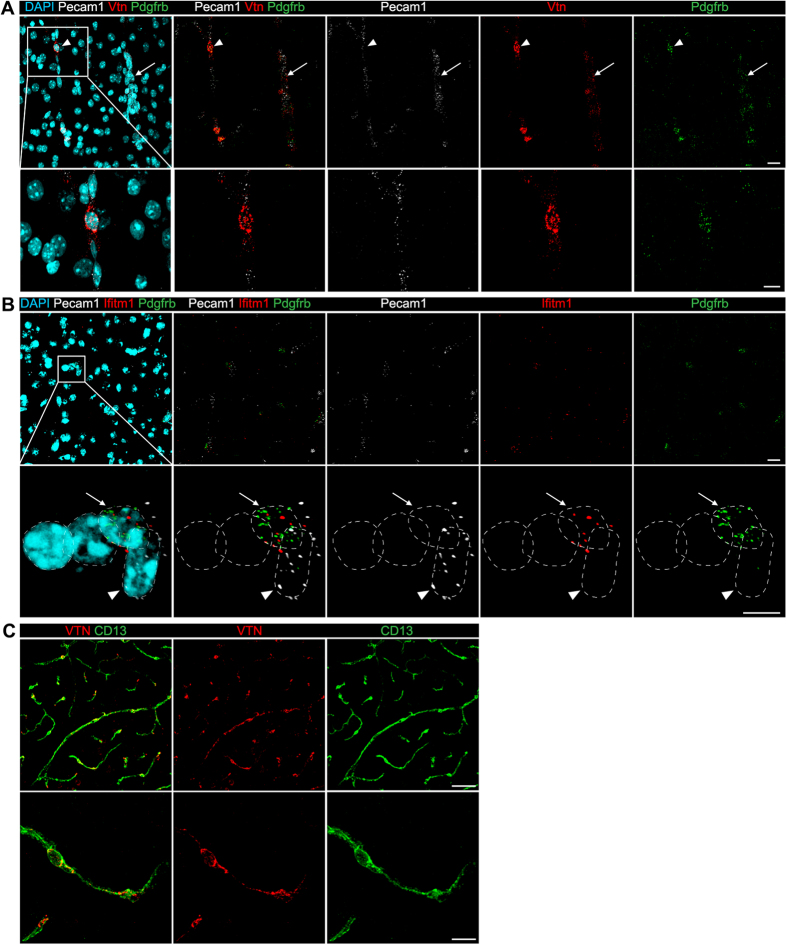
Validation of novel brain pericyte markers by *in situ* hybridization and immunohistochemistry. RNA *in situ* hybridization of 2-month old C57BL6 mouse cerebral cortex. (**A**) Vitronectin (*Vtn*, in red) is expressed in PC, identified by *Pdgfrb* expression (in green). While *Pdgfrb* expression is restricted to the cell body, *Vtn* can be found also in PC processes. Arrowhead points to a PC, arrow points to a vascular smooth muscle cell. In magnified inset is seen an overlap of *Pdgfrb* and *Vtn* expression in a PC. Cell nuclei are visualized by DAPI (in blue) and EC are visualized by *Pecam1* expression (in white). (**B**) Interferon induced transmembrane protein 1 (*Ifitm1*, in red) is expressed in PC, identified by *Pdgfrb* expression (in green). In inserts, cell nuclei are encircled with dotted lines. Arrow points to a PC nucleus, arrowhead points to an EC nucleus. Cell nuclei are visualized by DAPI (in blue) and EC are visualized by *Pecam1* expression (in white). (**C**) Immunohistochemical detection of vitronectin (in red) in PC (in green, CD13). The higher magnification image (C’) shows a strong expression of vitronectin in PC cell body and processes. The scale bars in top and lower panels are 20 μm (**A,B**), 50 μm (**C**) and 10 μm (**A,B** and C’), respectively.

Although we argue that the full 260-gene catalogue provides a conservative and reliable source of marker transcripts that distinguish mural cells including PC from EC in the brain, we chose two novel candidate markers for brain PC for validation by fluorescent *in situ* hybridization (ISH): Vitronectin (*Vtn*) and interferon induced transmembrane protein 1 (*Ifitm1*). These were selected based on their specificity for mural cells and mRNA abundance in the RNA-seq dataset from the present study. Both showed strong specificity; >9 fold enrichment for *Vtn* and >5 fold enrichment for *Ifitm1* in the FACS sorted *Pdgfrb-eGFP/NG2-DsRed* double positive cells compared with microvascular fragments. However, their abundance differed markedly; *Vtn* FPKM = 885; *Ifitm1* FPKM = 49. Accordingly, ISH detected *Vtn* mRNA at high abundance in PC surrounding capillaries, with mRNA detectable both in the cell body and PC processes (Fig. 4A, arrowhead). In these experiments the PC identity of single cells was determined by co-labeling for *Pdgfrb* (positive) and *CD31/Pecam1* (negative) mRNA. A specific *Vtn* signal was also detected in mural cells surrounding blood vessels with a wider diameter (arterioles or veins) but the signal in these cells was lower than in capillary PC (Fig. 4A, arrow). Immunohistochemical staining of brain slices using anti-VTN antibodies confirmed the ISH result (Fig. 4C), in that it co-localized with PC cell bodies and cellular processes. In accordance with the lower FPKM for *Vtn* in the RNA-seq analysis, *Ifitm1* ISH displayed a weak but specific signal in PC (Fig. 4B).

### Subcellular location of the brain mural cell-enriched genes

We mapped the 260 brain mural cell-enriched genes into their cellular locations using the CellWhere database^46^ (Table 3). This classification showed that 49 genes (19%) encode extracellular proteins, 98 genes (38%) encode membrane proteins, 67 genes (25%) encode cytoplasmic proteins, 30 genes (12%) encode nuclear proteins and 16 genes (6%) encode proteins with unclear/ambiguous cellular location. The proportion of the genes that encode extracellular and plasma membrane proteins is statistically significantly higher (Hypergeometric test) in the 260 mural cell-enriched (2–3 fold) than in any randomly selected gene list.

### Enriched pathway and interaction networks among the pericyte-enriched gene catalogue

We further mapped the brain mural cell enriched gene catalogue of 260 genes to the Kyoto Encyclopedia of Genes and Genomes (KEGG) pathways and identified a number of pathways that were significantly enriched (Table 4). Whereas most of these appear to reflect functions related to cell contraction and adhesion, perhaps expected from any mural cell population, the pathway *Neuroactive ligand-receptor interaction* may reflect some specific properties of brain mural cells and their role in the neurovascular unit. Transcriptional profiling of mural cells isolated from other organs than brain may shed light on this issue.

**Figure 5:**
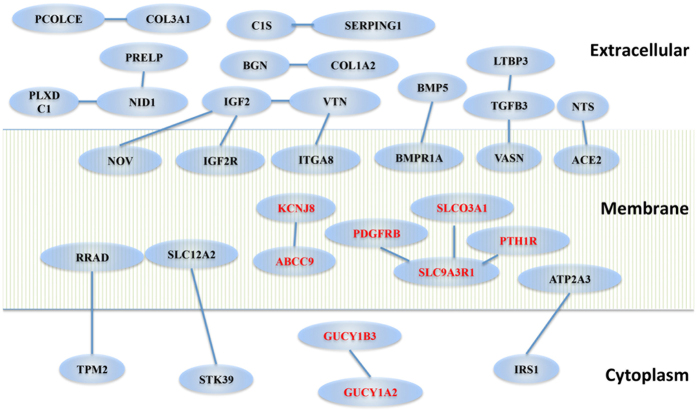
Protein interaction groups among brain mural cell-enriched genes. Proteins are represented with nodes and interactions between them are indicated with lines. The proteins are grouped according to their subcellular location (i.e cytoplasmic, membrane or extracellular). Proteins discussed in the text are indicated in red.

The Human Protein Reference Database (HPRD; www.hprd.org) contains manually curated protein–protein interaction data from published literature. To obtain information on protein-protein interactions within the 260-list, we mapped the corresponding human orthologues and extracted their protein-protein interaction information. This revealed over 20 interaction pairs and several interaction groups (Fig. 5). Among these, some proteins belong to the same family, such as GUCY1A2 (guanylate cyclase 1, soluble, alpha 2) and GUCY1B3 (guanylate cyclase 1, soluble, beta 3), or form ligand-receptor pairs, such as IGF2 (insulin-like growth factor 2) and IGF2R (insulin-like growth factor 2 receptor). Additionally, proteins with known functional connections were detected, such as KCNJ8 and ABCC9.

For soluble guanylate cyclases (GC), alpha and beta subunits interact to form the active enzyme. GC play a key cardiovascular role as targets of nitric oxide (NO), catalyzing the production of the intracellular signaling molecule cyclic guanosine monophosphate (cGMP)^47^. Homozygous *Gucy1b3* null mice die soon after birth, displaying gastrointestinal obstruction and hypertension phenotypes^48^. Soluble GC have been demonstrated in rat skeletal muscle PC^49^, but its enriched expression in brain PC have not been reported previously. It is possible that GUCY has a role in regulation of brain vessel diameter. However, glutamate evoked capillary dilation mediated by PC requires nitric oxide but is independent of GC^11^.

SLC9A3R1 (solute carrier family 9, isoform A3, regulatory factor 1, also known as EBP50, NHERF) is a Na(+)/H(+) exchanger regulatory factor. SLC9A3R1 binds PDGFRβ and potentiates its activity *in vitro*^50^. SLC9A3R1 interacts with SLCO3A1 (solute carrier family 21 member 11, namely OATP-D) through its PDZ domain^51^ and binds to PTH1R (parathyroid hormone 1 receptor)^52^. The physiological relevance of these interactions is unclear, but may engage in signaling and molecular trafficking between PC and neighboring cells. Importantly, their functional roles in PC *in vivo* await further investigation.

KCNJ8 and ABCC9 jointly form an ATP/ADP-binding potassium channel^53^. We previously identified these two genes as brain PC markers in mouse^39^. Recently, mutations in any of the two genes were linked to Cantú Syndrome^54^,^55^, a genetic disease that manifests with multiple phenotypic abnormalities, including excess of hair growth, skeletal abnormalities and various cardiovascular problems^56^. The specific expression of KCNJ8 and ABCC9 in vascular mural cells, in particular brain PC, may suggest a role for these cells in the pathogenesis of Cantú Syndrome.

## Discussion

While vascular mural cells are undoubtedly heterogeneous, ranging from typical VSMC to typical PC, the definition of PC remains ambiguous. Several functions have been tied to PC, including regulation of developmental blood vessel stabilization^4^,^5^, blood flow^11^, vascular barrier formation and integrity^9^,^10^ and leukocyte trafficking^57^,^58^. PC have also been assigned a stem cells function in tissue repair and fibrosis^59^,^60^,^61^,^62^,^63^,^64^,^65^. Usually, these functions have been studied in single or few organs, and it is unclear whether they are general properties of PC. It is also not resolved whether PC constitute a homogenous cell population within each organ, or if there are subtypes with different functions. The latter has been suggested in the context of spinal cord injury^66^. Moreover, the identity of the type of mural cells that regulate blood-flow in the CNS remains debated^67^,^68^. Mural cells have several developmental origins^2^, but whether this translates into molecular or functional differences is not known.

Only a handful of PC markers are known, all of which are dynamic in their expression, and none of them is specific to PC. Adding further to the difficulties, anatomical location and morphological hallmarks are ambiguous criteria for PC identification, since several other cell types are tightly apposed to EC. The original defining characteristic of PC was that they are embedded within the microvascular BM. While this definition is still applied, it is problematic. Transmission electron microscopy (TEM) is usually required to reveal the BM-embedded nature of PC, but TEM is unsuited for quantitative or high throughput studies or in situations where the BM is immature or abnormal. A seemingly vascular BM-embedded cell might be sandwiched between neighboring vascular and non-vascular BMs. This may be the case for cells located within the Virshow-Robin space. For practical reasons, current PC identification therefore relies on a combination of morphological criteria and marker expression^2^. Commonly used markers include *Pdgfrb*^4^, *Cspg4* (NG2)^69^, *Des* (desmin)^5^ and *Anpep* (CD13)^2^, but several other markers have also been described, including *Rgs5*^70^, *Abcc9 (Sur2*)^39^, *Kcnj8 (Kir6.1*)^39^, *Dlk1*^39^, and *Acta2*^71^.

Here, we applied a double transgenic reporter approach in order to isolate and characterize the transcriptome of brain vascular mural cells. PDGFRβ and NG2 are both commonly used markers for the identification of PC by immunohistochemistry, and *NG2-DsRed* mice have previously been used to image PC^42^. As mentioned before, neither PDGFRβ nor NG2 are specific for vascular mural cells; PDGFRβ is also expressed by fibroblasts that are located in meninges and around larger blood vessels in the CNS^72^. We found *Pdgfrb-eGFP* expressed in neuronal cells in periventricular areas and *Cspg4*/NG2 by OPCs as expected, in addition to mural cells. Thus, the use of any single marker for FACS results in substantial heterogeneity, however, the double reporter was specific for mural cells. PC were uniformly strongly double positive for *Pdgfrb-eGFP* and *NG2-DsRed,* whereas VSMC labeling was heterogenous, with a proportion of the cells expressing little to no levels of *NG2-DsRed.* It is therefore possible that certain subtypes or sub-phenotypes of VSMC are not represented in our dataset. The double labeling approach for specific PC identification in brain was recently demonstrated using *Pdgfrb-Cre* and *NG2-Cre*-mediated reporter activation^73^, which provided a view of brain PC morphology and distribution that matches our data using *Pdgfrb-eGFP;NG2-DsRed* mice.

Previous studies have reported on mouse mural cell transcriptomes *in vivo*, each utilizing different approaches to achieve cell type specificity and different transcriptional profiling approaches^9^,^39^,^40^,^41^. This work includes two of our previously published microarray studies on mouse mutants (*Pdgfb*^−/−^, *Pdgfrb*^−/−^, *Pdgfb*^*ret/ret*^ and others) that genetically lack or have severely reduced PC numbers^9^,^39^, in which down-regulated transcripts in brain microvessels were considered candidate PC markers. A similar indirect approach was taken using brain EC isolates depleted from contaminating PC using anti-PDGFRβ antibodies^40^. A recent study provided preliminary insight into the mouse brain mural cell transcriptome through single cell RNA-seq^41^. It is reasonable to consider that each of these studies have their own advantages and limitations, especially with regard to contaminating cell types, but also considering the profiling methodology and depth of analysis. As listed in Table 2, potential PC-enriched genes were identified as down-regulated genes in the PC-deficient mice^9^,^39^. Expectedly, these two gene lists also include EC genes that are down-regulated secondary to PC deficiency, which likely explains a higher percentage of EC gene contaminations (22–27%) in those studies as compared with the other two studies (6–8%)^40^,^41^. For most of these studies it is important to consider that the data does not differentiate between PC and VSMC. The exception is the single cell RNA-seq study^41^, which provides preliminary insight into differences between PC and VSMC. However, due to the limitation in the number of presumptive VSMC (n=62) and PC (n=21) identified, the available sequence information is incomplete and noisy, duplicated word.

When comparing the results from all five mentioned studies^9^,^39^,^40^,^41^, a surprisingly limited overlap among mural cell-specific genes was observed. This may be explained by different development stages, physiological and pathological conditions (Table 2) and platform bias. We propose, however, that genes identified by at least two approaches (260 genes) should be considered a conservative list of mural cell enriched transcripts. This list includes all known markers for PC but also a large number of genes that have not been previously linked to mural cell biology. The reader should also be aware that some mural cell-specific genes, which are only expressed at certain time points, might be missed in this list. *In silico* analysis of functional annotations and protein interaction networks pinpointed several significantly enriched pathways and protein interaction groups. To further validate the mural cell-enrichment, we selected two genes, *Vtn* and *Ifitm1*, for ISH validation. Despite different gene expression levels, both genes were shown to display a distinctly mural cell-specific pattern, with the majority in PC, although expression was detectable also in the VSMC of larger vessels. From the previously published single cell RNA-seq data (Figure S10)^41^, these two genes also showed higher expression in PC compared with other vascular cells. The functional roles of *Vtn* and Ifitm1 in PC await further analysis.

In conclusion, our analysis provides the most comprehensive, yet conservative, catalogue of mouse brain mural cell-enriched gene transcripts available to-date. It sheds new light on the molecular composition of the brain mural cells, including PC, and their molecular signaling pathways and interaction networks. Our analysis suggests numerous new PC markers, of which two were validated by ISH and/or IHC, and provides a rich source for new hypotheses to be tested about PC biology in health and disease.

## Materials and Methods

### Animals and animal experiments

The following wild type and transgenic animals of either sex were used: C57BL6/J, *NG2-DsRed* mice^42^, *Pdgfrb-eGFP* mice (Gensat.org. Line name: Tg(*Pdgfrb-eGFP*)JN169Gsat/Mmucd). Animal experiment protocols were approved by the Stockholm North Ethical Committee on Animal Research (Permit number N16/12), the Uppsala Ethical Committee on Animal Research (Permit number: C224/12 and C225/12), and by the Cantonal Veterinary Office Zurich, Switzerland (ZH196/2014). All animal experiments were carried out in accordance with their guidelines.

### Antibodies and immunohistochemistry

Mice under full anesthesia were euthanized by transcardial perfusion with Hanks buffered salt solution (HBSS, cat. # 14025092, GIBCO) containing 5 U/ml heparin followed by 4% paraformaldehyde in phosphate buffered saline (PBS). Tissues were removed and postfixed in 4% paraformaldehyde for 4 hours at 4 °C. Immunohistochemistry was carried out on vibratome brain coronal sections (30 or 50 μm) or on whole isolated retinas, as described^9^. Briefly, tissues were blocked and permeabilized in PBS containing 1% bovine serum albumin and 0.5% TritonX-100 in PBS overnight at 4 °C. Tissues were then incubated in primary antibody solution, washed, and subsequently incubated in secondary antibody solution. Each step was carried out overnight at 4 °C. Sections were mounted in Prolong Gold Antifade reagent with DAPI (cat. # P36931; Invitrogen). The following primary antibodies were used: mouse anti-human α-smooth muscle actin (Cy3 conjugated - cat. # C6198, FITC conjugated - cat. # F3777, Sigma Aldrich); rat anti-mouse CD31 (cat. # 553370, BD PharMingen); goat anti-CD13 (cat. # AF2335, R&D Systems); rabbit anti-vitronectin (cat. # GWB-794F8F, GenWay); rat anti-mouse PDGFRβ (cat. # 14-1402, eBioscience). The fluorescently labelled secondary antibodies made in donkey or goat were purchased from Jackson ImmunoResearch.

### Microvascular isolation

After cervical dislocation, the brains of three *Pdgfrb-eGFP/NG2-DsRed* mice at postnatal day 6 (P6) were dissected out and divided into two sagittal halves, of which one was processed for microvascular isolation and the other half was processed for FACS analysis (see further). The tissue was chopped into small pieces with scissors, and incubated with 0.5 mg/ml of collagenase 2 (Sigma, C6885) in Dulbecco’s Modified Eagle’s Medium (DMEM, Life Technologies) for 10 minutes at 37 °C. Afterwards, the suspension was neutralized with an equal amount of DMEM with 20% fetal bovine serum (FBS, Life technologies) to block collagenase activity. The digested tissue was then filtered over a 70 μm mesh (BD Biosciences) to remove large undigested tissue pieces. After centrifugation, the pellet was resuspended in DMEM with 0.5 mg/ml heparin (Sigma) to prevent coagulation. The cell suspension was incubated with sheep anti-rat magnetic Dynabeads (Life technologies) bound to rat anti-mouse CD31 antibody (BD Pharmingen) for 30 minutes at room temperature to allow coating of microvascular fragments with beads. Afterwards, the microvascular fragments were separated from the rest of the tissue suspension with a DynaMag-2 magnet (Life technologies) and washed three times with DMEM containing 0.5 mg/ml of heparin. Finally, the microvascular fragments were lysed in RLT buffer and RNA was extracted with the Qiagen RNeasy micro prep kit according to the manufacturers protocol (Qiagen).

### FACS

From a sagittal half of the brain, a single-cell suspension was obtained for FACS analysis in the following way. The tissue was minced with scissors and incubated with DMEM containing 1 mg/ml of collagenase 2 at 37 °C for 5 minutes with vigorous shaking. The suspension was then mechanically dissociated by pipetting up and down 10 times with a p1000 pipette, followed by another incubation for 5 minutes at 37 °C. Afterwards, the collagenase 2 was neutralized in the suspension by addition of an equal volume of DMEM with 20% FBS. The cells were filtered over a 70 μm mesh and centrifuged to obtain a pellet, which was resuspended in DMEM containing 2% FBS. Cells positive for eGFP and DsRed were obtained by FACS using a BD FACSAria III (100 μm nozzle size, 20 psi sheet pressure, 488 nm and 561 nm excitation lasers for eGFP and DsRed, respectively) at the BioVis core facility at the Department of Immunology, Genetics and Pathology (IGP), Uppsala University. Cells were sorted straight into RLT lysis buffer and RNA was extracted with the Qiagen RNeasy micro prep kit according to the manufacturers protocol (Qiagen). The scatter plots were generated using FlowJo v10.0.8r1 (FlowJo, LCC) to illustrate the intensity of eGFP and DsRed channel of analyzed cells.

### RNA-seq library construction

RNA integrity and concentration of the FACS sorted cells and the isolated microvasculature was checked with the Agilent RNA 6000 pico kit and the Agilent 2100 Bioanalyzer (Agilent Biotechnologies). For conversion of the RNA to a cDNA library for Illumina sequencing, the SMARTer^®^ Stranded Total RNA Sample Prep Kit - Low Input Mammalian kit from Clontech was used according to the manufacturer’s protocol. The total RNA was depleted from rRNA and tRNA’s with the RiboGone- Mammalian kit (Clontech) prior to library construction. The samples were sequenced on an Illumina HiSeq 2500 sequencer at the SNP&SEQ sequencing facility (Science for Life laboratory (SciLifeLab), Uppsala sequencing node).

### *In situ* RNA hybridization

*In situ* RNA hybridization was performed using RNAscope technology (Advanced Cell Diagnostics) following the manufacturer’s protocol with minor modifications. Briefly, fresh-frozen brains were cut into 16 μm sagittal sections and mounted on SuperFrost Plus glass slides. After dehydration, slides were subjected to RNAscope Multiplex Fluorescent Assay. First, slides were incubated in Pretreat 4 for 20 min at RT. After that, RNAscope probes were hybridized for 2 h at 40 °C and the remainder of the assay protocol was implemented. The fluorescent signal emanating from RNA probes was visualized and captured using a Leica TCS SP8 confocal microscope (Leica Microsystems). All *in situ* hybridization images presented are 2D maximum intensity projections of ~3 μm z-stacks. According to the Advanced Cell Diagnostics, each mRNA molecule hybridized to a probe appears as a separate small fluorescent dot.

### RNA-seq data analysis

The sequencing libraries for three microvascular samples and three FACS sorted cells were pooled after indexing and ran on a single lane (paired-end sequencing, 125 bp reads, high-output mode). In each sample, over 25 million pair-end reads were sequenced. The reads were aligned to the Ensembl mouse gene assembly (NCBIM37) using Tophat2 software (version 2.0.4)^74^. The duplicated reads were removed using the picard tool (version 1.92, http://broadinstitute.github.io/picard/). To identify the genes significantly enriched in the PC samples as compared with microvascular samples, statistical tests were performed using the Cufflinks tool (version 2.2.1)^75^.

### Meta-analysis of mural cell-enriched genes

The potential mural cell-enriched genes were collected from four published studies, and also included the results from our current RNA-seq study on FACS sorted brain mural cells.

From our previously published Affymetrix microarray study using *pdgfb*^ret/ret^ mutant mice (NCBI GEO accession number GSE15892), the four *pdgfb*^ret/ret^ brain vascular samples were compared with controls. The raw array data were normalized using Bioconductor (www.bioconductor.org) GCRMA package (version 2.32.0) and the probe sets which had signals defined as present in at least half of the arrays were selected for further analysis. Significance Analysis of Microarrays (SAM) method (Bioconductor Siggenes package, version 1.34.0) was applied to identify the significantly differentially expressed genes. The affymetrix probe sets were mapped to the NCBI Entrez Gene using the Biocondcutor annotation package (mouse4302.db, version 2.9.0). From the analysis, 453 genes which are more than two fold down-regulated in the *pdgfb*^ret/ret^ as compared with controls were identified (False Discovery Rate <0.05).

As described previously^39^, 142 mouse UniGene Clusters were reported as more than two fold down-regulated in both *Pdgfb* and *Pdgfrb* mutant brain microvessels, compared with controls. They were mapped to 135 NCBI Entrez Genes and were used in the comparisons.

From the BBB transcriptome data performed by Daneman *et al*.^40^, two lists of brain PC and brain endothelial-enriched genes were reported. The affymetrix probe sets were mapped to the NCBI Entrez Gene using the Biocondcutor annotation package (mouse4302.db, version 2.9.0). In total, there are 175 brain PC-enriched genes and 1631 brain endothelial-enriched genes.

In the recently published brain single cell RNA-seq analysis, 155 genes were reported to be enriched in the brain mural cells. They were mapped to the NCBI Entrez Gene using gene symbol.

To compare the five data sets and visualize the result, we used the R code which we posted in the bioconductor community previously (https://support.bioconductor.org/p/19693/).

### Pathway analysis

Pathway analysis was performed using KEGGprofile package (version 1.2.0) in R software (www.r-project.org). It carries out Hypergenometic tests to identify the KEGG pathways that are over-represented among the 260 identified mural cell-enriched genes, and p < 0.01 was considered statistically significant and pathways with at least nine mural cell-enriched genes were selected.

## Additional Information

**Accession codes**: Our RNA-seq data have been deposited in NCBIs Gene Expression Omnibus (http://www.ncbi.nlm.nih.gov/geo/) with accession number GSE75668.

**How to cite this article**: He, L. *et al*. Analysis of the brain mural cell transcriptome. *Sci. Rep.* **6**, 35108; doi: 10.1038/srep35108 (2016).

## Supplementary Material

Supplementary Information

Supplementary Dataset 1

Supplementary Dataset 2

## Figures and Tables

**Table 1:** Gene expression of known pericyte markers in microvasculature and pericyte samples.

Ensembl ID	Symbol	Microvasculature (FPKM^a^)	Mural cell (FPKM^a^)	Enrichment fold^b^ (Mural cell/Microvasculature)
ENSMUSG00000035783	Acta2	58,2	726,2	12,5
ENSMUSG00000030247	Kcnj8	12,6	136,7	10,9
ENSMUSG00000024620	Pdgfrb	45,4	445,2	9,8
ENSMUSG00000056481	Cd248	35,9	341,2	9,5
ENSMUSG00000039062	Anpep	9,2	83,8	9,1
ENSMUSG00000026208	Des	8,9	81,0	9,1
ENSMUSG00000040856	Dlk1	7,2	64,7	9,0
ENSMUSG00000032368	Zic1	47,3	376,8	8,0
ENSMUSG00000030249	Abcc9	35,7	282,3	7,9
ENSMUSG00000026678	Rgs5	176,5	1302,3	7,4
ENSMUSG00000032911	Cspg4	39,7	257,3	6,5

Listed are RNA-seq expression values for the known pericyte markers.

^a^The expression quantification is given by FPKM (Fragments Per Kilobase Of Exon Per Million Fragments Mapped).

^b^The enrichment fold shows the ratio of FPKM values between PC and microvasculature samples.

**Table 2:** Some details of five studies on the brain mural cells transcriptome.

	Experimental model	Age	# of identified mural cell genes	# of identified mural cell genes after removal of endothelial genes	Endothelial genes (%)	Reference
1	Pdgfb^ret/ret^ mutant versus control	7–10 months	453	354	21,85%	Armulik *et al*.^9^
2	Pdgfb/Pdgfrb mutant versus control	E17.5	135	99	26,67%	Bondjers *et al*.^39^
3	Vasculature versus endothelium	P60-70	175	164	6,29%	Daneman *et al*.^40^
4	Single cell analysis of brain mural cells	P21-31	155	142	8,39%	Zeisel *et al*.^41^
5	*Pdgfrb-eGFP* and *NG2-DsRed* double positive mural cells	P6	856	788	7,94%	current study

Listed are details of four previously published studies and our current study. The following details are listed: the experimental model, the age of the mice, the number of total identified mural genes, the percentage of contaminated EC genes, the number of mural genes after removal of EC genes and references.

**Table 3:** Subcellular location of the brain mural cell-enriched genes.

Cellular location	# of genes	Gene symbols
Extracellular	49	Adamts15 Adamts16 Adamts8 Arhgap42 Aspn Bgn Bmp5 C1qtnf2 C1qtnf7 C1s Ccdc80 Chn1 Col1a2 Col3a1 Col5a2 Crispld2 Daam2 Efemp2 Egflam Emilin1 F2r Fstl1 Gpc6 Hspa12a Igf2 Il34 Lama2 Lgals1 Lipg Ltbp3 Nid1 Nodal Nov Ntn1 Ogn Pcolce Pdgfa Pgf Pla1a Prelp Ptn Pxdn Rarres2 Serping1 Serpini1 Sfrp2 Tnxb Vtn Wnt5b
Membrane	98	Abcc9 Ace2 Adcy6 Amigo2 Ano1 Ano4 Aoc3 Art3 Atp13a5 Atp1a2 Atp1b2 Atp2a3 Axl Bmpr1a Cacna1h Cadm4 Cap2 Cd248 Cdh11 Cpm Cspg4 Dlk1 Dmd Ednra Enpep Eps8 Evc Fap Fgfr3 Gfra2 Ggt1 Glrb Gpr20 Gpr30 Gprc5b Gpx8 Grm3 Higd1b Ifitm1 Igf2r Itga7 Itga8 Jam3 Kcne4 Kcnj8 Kcnmb1 Kirrel Lhfp Lin7a Marveld1 Mboat2 Mcam Mrvi1 Naalad2 Notch3 Npy1r Olfr558 Olfr78 P2rx1 P2ry14 Pcdh18 Pde3a Pdgfrb Pla2r1 Plekha2 Plxdc1 Plxdc2 Plxnb1 Prkar1b Ptger3 Pth1r Rasl12 Rftn2 Ror1 Rrad S1pr3 Samd4 Scn2b Sdc2 Sema6d Sgca Sh2d3c Slc12a2 Slc16a12 Slc19a1 Slc30a10 Slc38a11 Slc6a20a Slc7a2 Slc9a3r1 Slco3a1 Steap4 Susd2 Sytl2 Tnfrsf21 Trpc1 Vasn Vstm4
Cytoplasm	67	Acta2 Adap2 Adrbk2 Aif1l Ankrd6 Arhgap10 Arhgef25 Cald1 Casq2 Cnn1 Cryab Ctnna3 Dact1 Dbndd2 Ddit4l Dennd2a Des Dgkb Dtx3 Fads3 Fam101a Fam20a Farp1 Filip1l Flna Frk Gucy1a2 Gucy1a3 Gucy1b3 Inpp4b Irs1 Lmcd1 Lmod1 Myh11 Myl9 Mylk Myom1 Ndrg2 Nts Palld Parva Pde1a Pde5a Pde8b Pdlim1 Pdlim3 Pid1 Pitpnc1 Plce1 Plcl1 Ppp1r14a Ptpn9 Rbms3 Rcn3 Rerg Rgs4 Rgs5 Sept11 Sept8 Stk39 Stmn2 Tagln Tgfb3 Tpm1 Tpm2 Uchl1 Wtip
Nucleus	30	Alx3 Arvcf Ccnd2 Ctdspl Etv1 Foxd1 Foxs1 Hey2 Heyl Hic1 Hspa2 Kctd1 Lbh Mustn1 Myocd Nr2f1 Nr2f2 Nrip2 Pawr Prickle2 Prrx1 Rasl11a Rgs7bp Runx1t1 Tbx15 Tbx18 Tbx2 Uba2 Vgll3 Zic1
Unclear	16	1110059G02Rik 1500009L16Rik 6330403L08Rik Arhgef17 AW011738 Fam107b Gm13889 Itga10 Kctd15 Klhl23 Mn1 Rbpms2 Samd5 St5 Susd5 Tbc1d2b

The identified 260 brain mural cell-enriched genes are classified into subcellular locations. The gene symbols and the total number of genes in each category are listed.

**Table 4:** Enriched signalling pathways in brain mural cell-enriched genes.

Pathway name	p value	# of gene	Gene symbols
Focal adhesion	2,72E-10	17	Ccnd2 Col1a2 Col3a1 Col5a2 Flna Itga10 Itga7 Itga8 Lama2 Myl9 Mylk Parva Pdgfa Pdgfrb Pgf Tnxb Vtn
Vascular smooth muscle contraction	2,10E-09	13	Acta2 Adcy6 Cald1 Ednra Gucy1a2 Gucy1a3 Gucy1b3 Kcnmb1 Mrvi1 Myh11 Myl9 Mylk Ppp1r14a
Dilated cardiomyopathy	4,00E-09	11	Adcy6 Des Dmd Itga10 Itga7 Itga8 Lama2 Sgca Tgfb3 Tpm1 Tpm2
ECM-receptor interaction	3,28E-08	10	Col1a2 Col3a1 Col5a2 Itga10 Itga7 Itga8 Lama2 Sdc2 Tnxb Vtn
Calcium signaling pathway	7,42E-06	11	Atp2a3 Cacna1h Ednra F2r Mylk P2rx1 Pde1a Pdgfrb Plce1 Ptger3 Trpc1
Regulation of actin cytoskeleton	9,86E-04	9	F2r Fgfr3 Itga10 Itga7 Itga8 Myl9 Mylk Pdgfa Pdgfrb
Neuroactive ligand-receptor interaction	1,67E-03	10	Ednra F2r Glrb Grm3 Npy1r P2rx1 P2ry14 Ptger3 Pth1r S1pr3

KEGG pathways enriched in the 260 brain mural cell-enriched genes. Statistical test’s p value, gene number and gene symbols are listed.
