# Supplementary material for: Analysis of the brain mural cell transcriptome
Source: Sci Rep. 2016 Oct 11;6:35108. doi: 10.1038/srep35108 (PMC5057134; doi:10.1038/srep35108)
Supplement: Supplementary Information [file srep35108-s1.pdf]

Supplemental Information

Analysis of the brain mural cell transcriptome

Liqun He, Michael Vanlandewijck, Elisabeth Raschperger, Maarja Andaloussi Mäe, Bongnam Jung, Thibaud Lebouvier, Koji Ando, Jennifer Hofmann, Annika Keller, Christer Betsholtz

Table S1. The five brain mural-enriched gene lists.

Listed are the detailed gene lists from the five studies (column C-G), with "1" and "0" to represent if the genes were identified or not by the corresponding studies, respectively. The genes were sorted by the FPKM values (from the current RNAseq study) in column H.

Table S2. The selected 260 brain mural cell-enriched gene catalogue.

Listed are the identified 260 brain mural cell-enriched genes. The genes were sorted by the FPKM values as in Table S1.
